# Battery-free head orientation measurement using passive RFID tags

**DOI:** 10.1017/wtc.2024.26

**Published:** 2025-02-17

**Authors:** Jeyeon Jo, Heeju T. Park

**Affiliations:** 1 Department of Textiles, Merchandising, and Interiors, University of Georgia, Athens, GA, USA; 2 Department of Human Centered Design, Cornell University, Ithaca, NY, USA

**Keywords:** sensors, soft wearable robotics, monitors, design

## Abstract

Real-time measurement of head rotation, a primary human body movement, offers potential advantages in rehabilitating head or neck motor disorders, promoting seamless human–robot interaction, and tracking the lateral glance of children with autism spectrum disorder for effective intervention. However, existing options such as cameras capturing the entire face or skin-attached sensors have limitations concerning privacy, safety, and/or usability. This research introduces a novel method that employs a battery-free RFID tag-based wearable sensor for monitoring head orientation, as a substitute for the existing options like camera. By attaching a pair of passive RFID tags to the front of the head at a specific distance from each other, the signal strength of each tag within the pair differs based on the discrepancy in distance from the RFID reader caused by head rotation. Important parameters including distance between the tags, distance from the reader, and tag types, are investigated to suggest optimal sensor design. In tests involving random head rotations by 10 healthy adults, there was a significant correlation between the orientation of the head and gaze in the yaw direction and the differences in signal strength from the sensor pairs. The correlation coefficients ( 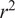 ) were satisfactory, at 0.88 for head and 0.83 for left eye pupil orientations. However, the sensor failed to estimate pitch rotations for head and gaze, due to the insufficient vertical spacing between the tags. No demographic factors appeared to influence the results.

## 1. Introduction

Head rotation is a part of fundamental human movements, serving as a primary means of social interaction and a source of comfort (Langton, 2000). In the medical field, the analysis of head motion is a crucial aspect of diagnosing and rehabilitating individuals with head and neck motor disorders (Ali et al., 2021; Mihajlovic et al., 2018; Yamanobe et al., 2022). Head motor impairments are also important signals for diagnosing and monitoring developmental disorders (Flanagan et al., 2012; Raya et al., 2011; Saavedra et al., 2010). Head orientation often serves to estimate the gaze or the accompanied social attention (Bizzi, 1974; Jiang et al., 2019; Lee et al., 2011; Sidenmark and Gellersen, 2019). Head orientation determines the field of view and can often represent eye gaze as an easier and more affordable method (Blattgerste et al., 2018; Renner and Pfeiffer, 2017; Špakov et al., 2019). Lateral glance, defined as looking at objects out of the corners of the eyes without head and gaze orientation alignment, is one of the most frequently observed atypical visual behaviors among the children with autism spectrum disorders (ASD) (Coulter, 2009; Mottron et al., 2007). One of the biggest consequences of this lateral glance is a challenge in social interactions, as maintaining appropriate eye contact is an essential skill in building relationships (Hellendoorn et al., 2014; Hustyi et al., 2023).

Systems connecting the head pose and the attention provide seamless interactions between the users and the robot agents (Tamaru et al., 2022; B. Zhang et al., 2014) or make the vehicles ready for upcoming direction changes for the drivers or wheelchair users (Takahashi et al., 2011; Zhao et al., 2017). Mouse cursor control through head movements for individuals with tetraplegia or cerebral palsy can be an alternative interaction technique to gaze-based mouses (Velasco et al., 2017; Williams and Kirsch, 2016). Head orientation is a big interest in virtual reality to maintain the coordination between the head movement and the camera view while minimizing any perceptual artifact (LaValle et al., 2014).

In order to identify head motion, inertial measurement units (IMUs) and cameras are the most commonly used approaches. Recent advances in computer vision especially have made it possible to achieve head or gaze tracking using cameras (Chang et al., 2021). Surveillance camera-style systems are popular type of gaze tracker, but continuously capturing the entire face raises privacy concerns (Bowyer, 2004; Tepencelik et al., 2021; Tobii, 2023). Alternative wearable methods have been introduced such as LiDAR, electromyography (EMG), microphones, or radiooculogram (Kamoshida and Takemura, 2018; Salinas-Bueno et al., 2021; Severin, 2020; Williams and Kirsch, 2008; Zhang and Kan, 2022), but most of them compiled electronic components that are often rigid, thick, and inflexible. They also require a power source and microcontrollers, increasing the weight and rigidity of the whole system.

Passive RFID (radiofrequency identification) tags are a suitable alternative for creating a soft, comfortable, lightweight, and low-cost wearable head motion sensor with wireless communication capability (Kiourti, 2018; Luo et al., 2020). Though it has not been used for the head orientation measurement, passive RFID tags have been introduced to track human body movements such as touch, joint bending, and pressure (Jin et al., 2018; Jo and Park, 2021; Li et al., 2016). Furthermore, its sensing capabiity toward temperature, and humidity increases its potential for the wearable applications (Meng and Li, 2016; Nath et al., 2006). When designing a wearable RFID-based sensor, it is important to consider the radio wave absorbance by body location, distance from the reader, accompanying body motions, and obstacles between the reader and the tags (Manzari et al., 2012), because it requires an RFID reader near the tags as a power source. As radio waves are susceptible to the environmental factors listed above, most applications using RFID are based on conditions where the RFID tag or user is relatively less active, such as sleeping. (Sharma and Kan, 2018a).

**Figure 1.**
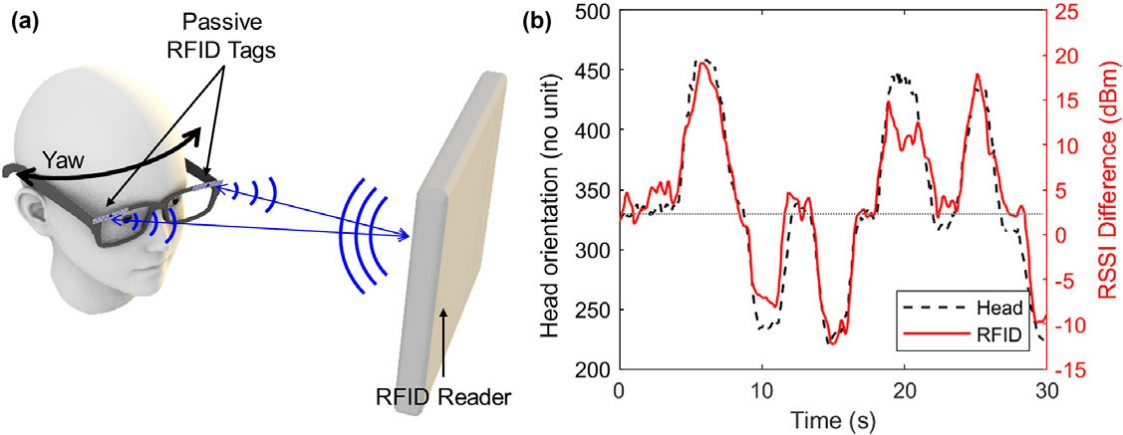
Passive RFID tag-based head orientation sensor. (a) Sensor design, (b) sensor response to random head orientations. The yaw angle of the head orientation is represented by the *x*-coordinates of the direction of the head pose projected onto a 2D plane parallel to the RFID reader. The range of values for yaw is 0–720. The horizontal thin dotted line at the center of the plane represents the direction of looking straight ahead on the screen.

This study aims to develop a soft and battery-free wearable sensor based on passive RFID tags to monitor the wearer’s head orientation, replacing existing powered sensors or vision-based systems such as cameras. In this study, we assumed that the user is watching a screen while maintaining the gaze at its center, so that any head turning leads to a misalignment of the head with the direction of the screen. The sensor uses pairs of passive RFID tags embedded in eye glasses, head pieces, and/or a face mask to track the wearer’s head movements while sitting in front of a screen with an RFID reader (Figure 1a). To verify the performance of the developed sensor, the random head orientation was tracked by the sensor and compared to the camera-based recordings in terms of correlation (Figure 1b). Additionally, this paper demonstrated the effectiveness of a pair of RFID tags attached to the frontal side of eyeglasses when used with an RFID reader-embedded laptop.

## 2. Sensor design

### 2.1. Sensing principle

**Figure 2.**
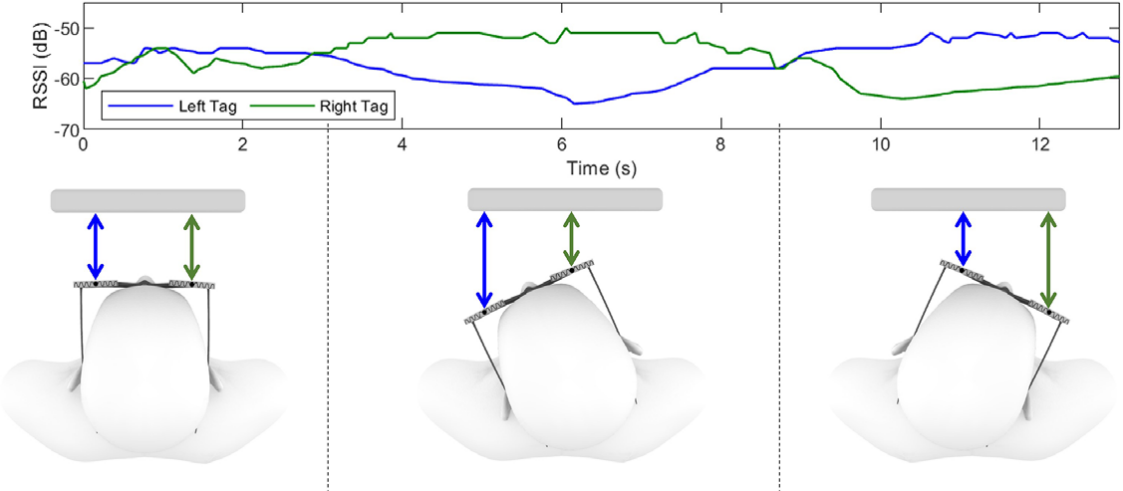
Sensing principle. The color of the lines in the plot corresponds to the arrow color in the illustrations.

The strength of the backscattered signals depends on the distance between the radio wave source and the tags according to the Frii’s free space loss: 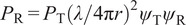 , where 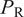 and 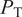 are the strength of the signal of the receiver and transmitter, respectively. 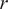 represents the distance between the transmitter and the receiver, 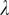 is the signal wavelength, and 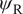 and 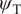 represent the gains of the receiver and transmitter antennas, respectively (Bolic et al., 2010). Passive RFID systems derives the electromagnetic field generated by the reader to operate the tag. Therefore, the received signal strength indicator (RSSI) can be described as follows: 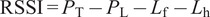 , where 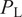 is path loss, 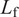 is additional losses such as multipath or antenna misalignment, and 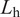 is harvesting loss from the conversion of the received energy to usable power in the tag. While 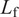 and 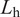 depend on the environment and the tag specifications, 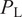 can be approximated as follows: 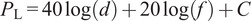 , where 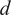 is the tag-reader distance, 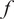 is operating frequency, and 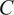 is constant related to the environmental factors such as speed of light. The current sensor uses a pair of passive tags, one on each side of the forehead (e.g., at each end of the foreside of the eyeglasses, as shown in Figure 1a), to track head rotation in the transverse plane (yaw – left and right). When the wearer turns their head to the left, the RFID tag on the left side moves farther from the reader, while the distance on the other side gets closer. This results in a decrease in the received signal strength indicator (RSSI) of the left tag and an increase in the signal strength of the right tag (Figure 2). In this sensor, the assumption is that the user will be continuously looking at the screen of an RFID reader-embedded device such as laptop. As the horizontally long form factor has been widely adopted for a long time (e.g., eyeglasses), this sensor basically aims to measure yaw rotations. However, the same principle can be applied to measure pitch on the sagittal plane (up and down) when a pair of tags are arranged vertically on the head. Therefore, we placed two pairs of RFID tags, one pair on the top and the other pair on the bottom edge of a pair of glasses. This allowed this study to primarily examine sensor performance in yaw while reducing noise by averaging the two pairs, as well as explore the potential for pitch movement.

To understand the behavior of the sensor through related parameters and to optimize performance, a pair of RFID tags were attached to the top left and top right corners of a pair of safety glasses. After the glasses were placed on a manikin’s head, tag readings were collected using an RFID reader (Speedway Revolution R220, Impinj, WA, USA) with an antenna (S9028PCR, Rfmax, NJ, USA). The RFID reader used in this study reported the RSSI with a resolution of 1 dBm across a range of 0 to −80 dBm. We rotated the manikin head so that the direction of the head and the reader were − 60, −45, −30, −15, 0, 15, 30, 45, and 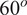 respectively, and collected the sensor reading for 5 seconds for each angle. The detailed setup is described in the following subsections.

### 2.2. RSSI difference by distance from reader

**Figure 3.**
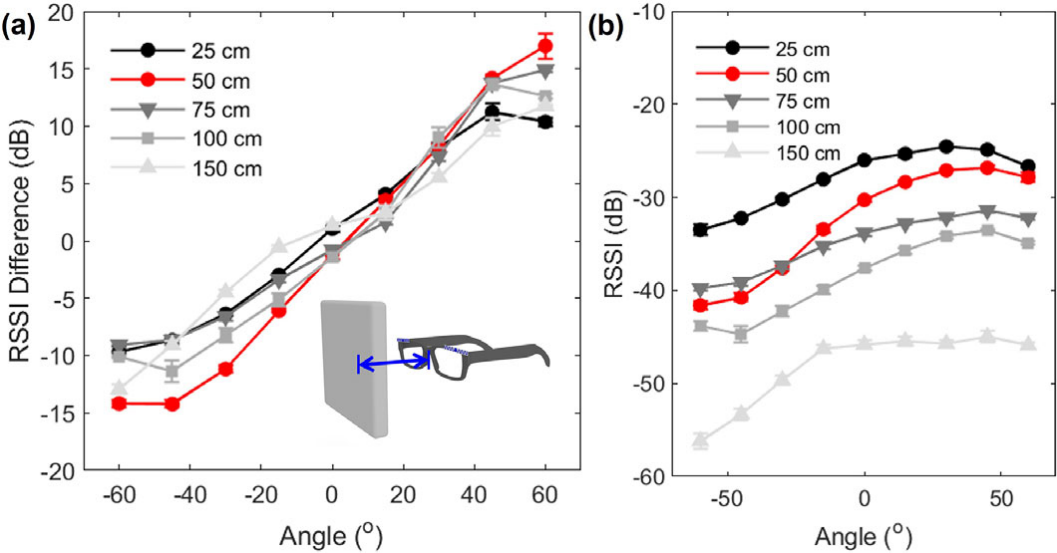
Sensor performance by distance from the RFID reader. (a) Difference in RSSI based on the distance from the reader and (b) mean RSSI of the left tag. The error bar indicates the standard error, and the red line plot represents the selected setting for the user evaluation study.

Consumer electronics with a screen can embed an RFID reader. However, the distance between the sensor and the user’s head varies depending on the type of the reader. For instance, smartphones are closer to the user’s eyes than televisions. To investigate the impact of the distance between the RFID reader and the tags, we rotated the sensor-worn manikin head from 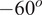 to 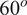 in yaw at distances of 25, 50, 75, 100, and 150 cm respectively, based on the various scenarios including those involving smartphones, tablets, laptops, and televisions. The sensor signal at 200 cm in a normal home environment was too weak to be included in the analysis. The sensor exhibited a distinct RSSI difference between the tags on each side when they were 50 cm away from the reader, which represents a common distance for laptop or tablet usage (Figure 3a). As anticipated, the sensor performance indicated by the difference in RSSI decreased as the distance from the RFID reader increased. The sensor was still able to detect angular changes at distances of 100 and 150 cm, but the low mean RSSI suggests that these distances may not be optimal for stable and continuous sensing (Figure 3b).

### 2.3. RSSI difference against distance between tags

**Figure 4.**
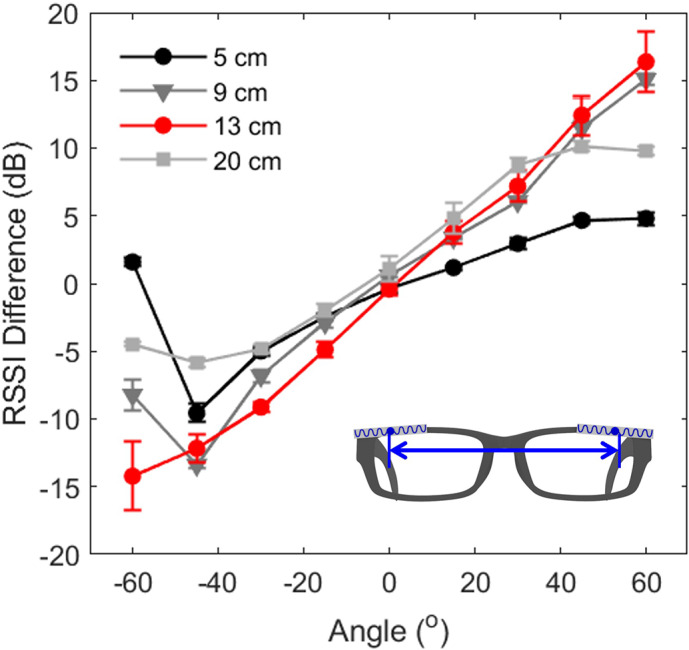
Sensor performance based on the distance between the tags. The error bar indicates the standard error, and the red line plot represents the setting selected for the user evaluation study.

The distance between the two tags can affect sensor performance as it determines the changes in the distance of each tag from the reader. To verify this, we have tested the available distances between the tag centers within a normal glass form factor. The results are as follows: 5 cm (right next to each other), 9 cm (in the middle of the lenses), 13 cm (left and right corner), 20 cm (on the legs). According to the results, when the center-to-center distance of the two tags was 13 cm, the RSSI difference changed linearly within the 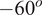 to 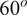 angle range in terms of the reader (Figure 4). Placing the tags on each leg of the glasses did not improve performance, even at the longest distance of 20 cm (measured on the surface, not linearly). Closer distances resulted in reduced RSSI difference due to smaller differences in distance from the reader.

### 2.4. RSSI of different RFID tag types

**Figure 5.**
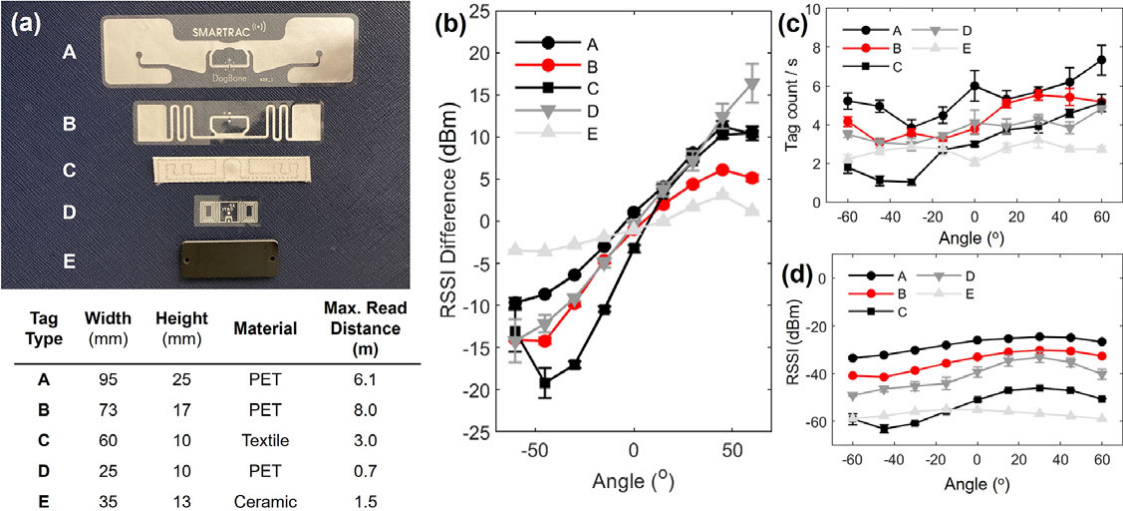
Sensor performance by type of passive RFID tag. (a) Details on the tags used in the study, (b) orientation sensing performance by tag, (c) mean tag counts per second by tag, and (d) mean RSSI by tag. The error bar indicates the standard error, and the red line plot represents the selected setting for the user evaluation study.

This study investigated five off-the-shelf RFID tags, including three made of PET (polyethylene terephthalate), one made of textile, and one made of ceramic (Figure 5a). The results showed that the signal strength of passive RFID tags is dependent on the dimension, shape, and material of the antenna. Tag C, which consisted of a canvas textile and a conductive thread antenna, exhibited the largest RSSI difference between the two tags overall (Figure 5b). Meanwhile, PET inlay-style tags (A, B, and D), which are the most common in the market, outperformed the textile tag in tag counts and RSSI (Figure 5c and 5d). The ceramic-encased tag E did not yield any favorable results. Tag A had the highest RSSI and tag count due to its larger dimensions, but its RSSI difference was moderate, and its size may not be suitable for eyeglasses or headpieces. The user evaluation implemented tag B with fair RSSI difference, signal strength, and appropriate dimensions.

## 3. Evaluation

### 3.1. Experimental protocol and data processing

**Figure 6.**
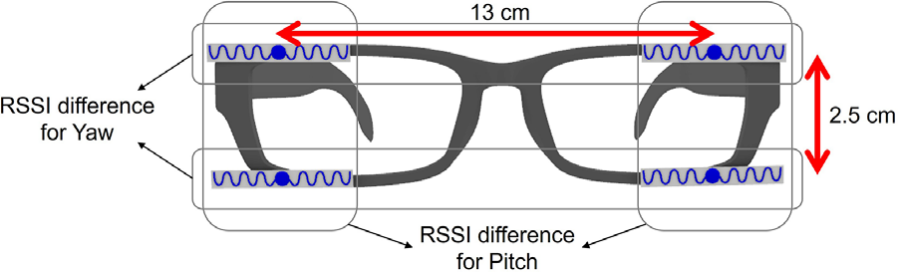
Sensor used in the experiments. Two pairs of RFID tags were attached to each corner of a pair of glasses to track two rotations (yaw and pitch) and to reduce noise.

Ten healthy adults (7 females and 3 males) with an average age of 25.5 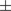 5.5 years and an average height of 168.3 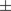 9.5 cm participated in the experiment. According to the experiment protocol approved by the Institutional Review Board (IRB), the participants wore safety glasses with four RFID tags (top-left, top-right, bottom-left, and bottom-right) attached at each corner. Having two pairs of RFID tags allowed not only to reduce RSSI noise due to multipath but also to examine two rotations (yaw and pitch). While the distance between the left and right tags was 13 cm based on results depicted in Figure 4, the glasses used in this study could only accommodate the maximum vertical distance of 2.5 cm (Figure 6). They sat on a chair in front of a desk, and an RFID antenna (S9028PCR, Rfmax, NJ, USA) connected to an RFID reader (Speedway Revolution R220, Impinj, WA, USA) was positioned above the laptop screen on the desk, approximately 50 cm away from their face. The laptop’s built-in webcam is located at the top of the screen, directly below the RFID reader antenna.

The current sensing principle requires the RSSI from the four tags to calculate the difference within each pair at the moment. For example, for yaw, the difference between the top-left and top-right tag readings and the difference between the bottom-left and bottom-right tag readings simultaneously were calculated. However, the RFID reader utilized in this study can only read one tag at a time, resulting in missing values when the RSSI of one tag is recorded. To address this issue, missing values were filled through linear interpolation in Matlab using adjacent data (Matlab, n.d.). The average RSSI differences from the two pairs of tags were smoothed using the moving-average method (window = 5). In the same way, the RSSI difference between the top-left and bottom-left, and the difference between the top-right and bottom-right were used for the pitch rotation.

To verify the performance of the current sensor, the user’s face was captured by the laptop camera at a rate of 30 Hz during the tasks. MediaPipe (Developers, n.d.), a computer vision Python library, was used to identify landmarks on the face. As the camera projects the head orientation onto the two-dimensional screen, the orientation data is collected as *x* and *y* coordinates instead of angles (Aflalo, 2022). This representation was suitable for this study, as the examination was conducted with only one direction at a time (yaw or pitch).

Participants were asked to perform two tasks. First, participants were instructed to randomly but continuously rotate their head for 1 min while fixating their gaze on the laptop screen. Following three repetitive sessions of this task, participants were asked to use the laptop for 5 min without any specific instructions.

The data from our sensor (i.e., the RSSI differences) were compared to the head orientation data from the camera by correlation in both yaw and pitch. For yaw, the main interest, we also performed correlation analysis with the projected direction of the eye pupils and the distance between the corners of the eyes (near the center) and the pupil. This allowed us to explore the potential for gaze tracking, as well as the possibility of monitoring discrepancies between head orientation and gaze (e.g., lateral gaze in children with ASD). They are labeled to as “Eye Pupil” and “Pupil-Eye Corner” in the following figures in this paper.

### 3.2. Results

**Figure 7.**
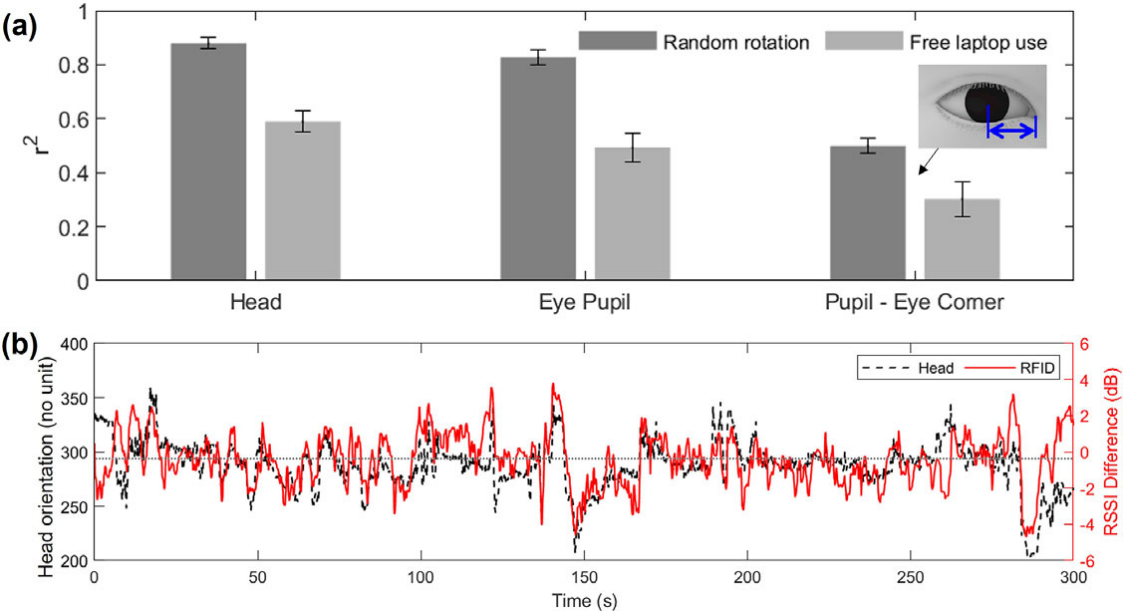
Evaluation results. (a) Mean correlation coefficient between the sensor and the head, eye pupil, and distance between the eye pupil and the eye corner during random head rotations. The error bars represent the standard error. (b) Head orientation from the vision and the sensor during 5-min free laptop usage. The yaw angle of the head orientation is represented by the *x*-coordinates of the direction of the head pose projected onto a 2D plane. The range of values for yaw is 0–720. The center of the screen is indicated by a horizontal gray dotted line, representing the direction of looking straight ahead.

In general, the sensor showed a lower RSSI on the head (−47.25 dBm) compared to the laboratory test based on the manikin head (−33.77 dBm) when the heads were aligned with the reader. The various materials in the human body (water, tissue, etc.) tend to absorb or scatter the RF signals from the transmitter compared to the synthetic materials of the manikin, which may reduce the strength of the received signals in both the tag and the reader. The sensor based on passive RFID tags demonstrated a strong correlation with both head and eye pupil movements when the user randomly rotated their head (Figure 7a). Specifically, the sensor had a correlation coefficient ( 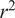 ) of 0.88 with head pose and 0.83 with left eye pupil movement. However, the distance between the pupil and the eye corner showed a relatively lower coefficient ( 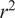 = 0.50), indicating a limitation in the current sensor system’s ability to track gaze rather than just head pose.

Meanwhile, the sensor’s performance during free laptop usage was not as impressive as during random, slow, and continuous head rotations. The correlation coefficient ( 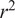 ) between the sensor and the head, left eye pupil, and the distance between the pupil and the eye corner were 0.59, 0.49, and 0.30, respectively. During the free laptop usage, there was less head movement compared to the intended and continuous head movement. Additionally, the noises became more distinguishable (Figure 7b). The lower signal-to-noise ratio (SNR) during the free laptop usage (−1.32 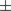 6.32 dB) compared to the intended movements (9.90 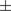 6.81 dB) confirms the higher influence of the noises.

**Figure 8.**
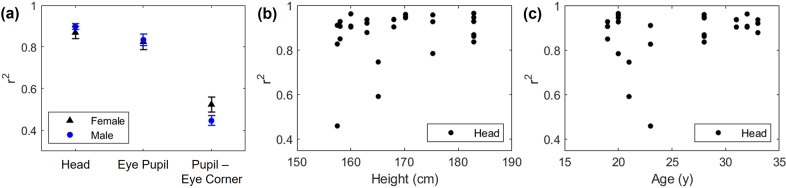
Correlation coefficient between the sensor and the ground truth by (a) sex, (b) height, and (c) age of the participants.

The demographic factors of sex, age, and height did not significantly influence or relate to the performance of the current sensor (Figure 8). The Wilcoxon rank sum test, a nonparametric method, did not find any significant differences (*p* > 0.05) between the two sexes. Similarly, the correlation tests of height and age with the correlation coefficients of the sensor did not yield any significant results (*p* > 0.05, respectively).

**Figure 9.**
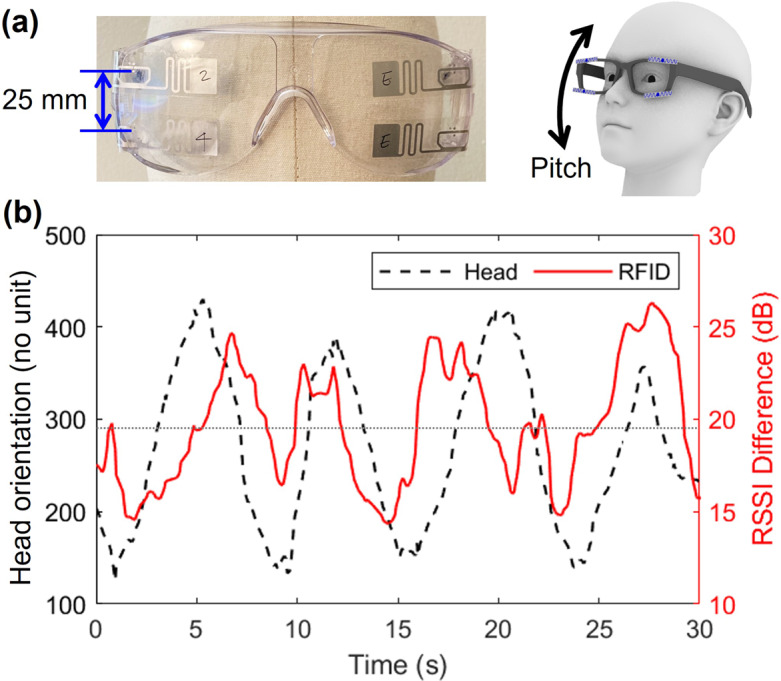
Measurement for pitch rotation. (a) The prototype with vertically arranged tags to measure head rotation in pitch, (b) head movements in pitch and the RSSI difference between the vertically arranged tags. The pitch angle of the head orientation was represented by the *y*-coordinates of the direction of the head pose projected onto a 2D plane parallel to the RFID reader antenna. The range of values for pitch is 0–480. The center of the screen is indicated by a horizontal gray dotted line, representing the direction of looking straight ahead.

The glasses-type sensor currently contained two pairs of RFID tags to reduce the vulnerability of RSSI of the passive tags against the multipath effect, which allowed it to explore the application of the same rotation sensing capability to measure head rotation in the pitch direction. This is particularly relevant for children with ASD who tend to look downwards (Noris et al., 2012). However, the distance between the vertically arranged RFID tags was only 25 mm (Figure 9a), which was too small to create a noticeable difference in the distance from the reader caused by head pitch rotation. This resonates with the poor performance of the pair that were too close to each other in Figure 4. Although the experiment detected a clear trend in RSSI (Figure 9b), it could not confirm that the difference represented head or gaze movements due to the low correlation between the RSSI readings and the random and continuous head rotation ( 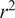 = 0.35). Furthermore, the correlation between the sensor readings and the head movements during free laptop usage was even lower ( 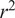 = 0.24).

## 4. Discussion

This study presents a soft and battery-free wearable sensor based on passive RFID tags to detect the head orientation of the wearer. The sensor showed a high correlation with camera-based head orientation tracking, demonstrating the potential to replace vision-based systems with the new battery-free sensor. The system had a higher sensing capability at a distance of 50 cm from the reader (Figure 3), making it more suitable for tablet or laptop usage rather than for smartphones or televisions. Although the current study used PET film-based tags for eyeglasses, textile tags could be a viable alternative when implementing the system into other accessories, such as headpieces or caps, due to their superior wearability and ease of maintenance. Meanwhile, the free laptop usage scenario showed much less impressive performance compared to the random and continuous rotation due to the noise during the static condition. This is a critical limitation that needs to be overcome in future studies, as the user scenario in this study required stationary media viewing, which does not involve much head movement. More pairs of tags than two as well as a faster sampling rate will help to reduce the noise when the wearer does not move the head much. Regarding the high correlation between the eye pupil and the sensor, it may mean that the participants moved the gaze following the head movements, even though we asked them to fixate on the center of the screen. This should be due to the natural tendency to move the eyes in the direction of the head, demonstrating the potential of the current sensor to capture gaze movements. Nevertheless, it is also possible that it was a limitation of the camera-based system not to track gaze appropriately, which needs to be investigated in future studies. Though pitch rotation measurement was not successful with the vertically arranged tags in the current prototype (Figure 8), the other wearable devices that cover a larger area of the face, such as masks or VR headsets, may be able to provide enough distance for pitch direction sensing. With that, the head circumference of children with ASD should also be carefully considered in the product specifically designed for them, as their head size tends to be smaller than that of adults but bigger than that of typically developing children (Sacco et al., 2015).

The current sensor can be used to optimize treatments for populations with above-neck mobility needs, such as those with neck disk or in rehabilitation. Additionally, the sensor can be applied to evaluate sleeping quality based on head pose and movements, which can be useful for developing sleeping masks (Sharma and Kan, 2018b). The current sensor’s head rotation measurement capability can be utilized in entertainment systems for VR or AR experiences, enhancing user interaction with physical environments. For instance, cardboard 3D glasses can maximize the battery-free motion sensing capability of the current system for improved interactions. As a single RFID reader can detect an unlimited number of tags within its detectable range, it is possible to provide this experience to multiple users who are looking in the same direction, such as in a museum, theater, or classroom (Jiang et al., 2019). Similarly, viewing media on a tablet or monitor while seating can align head, gaze, and screen. In this sense, although the sensor was not successful in tracking the eye pupil-eye corner distance representing side looking, head orientation monitoring itself may be able to contribute to tracking or diagnosing lateral gaze behavior in children with ASD.

The rotation sensing capability without a battery can be applied not only to wearable devices but also to other applications related to human–computer interaction. For instance, an interactive screen with an RFID reader can respond to a product with a pair of RFID tags when the customer rotates or shakes the product in retail stores. This enables the screen to track the orientation of the product and display it on the screen like a magic mirror, without using a camera. In an educational setting, RFID tag-embedded toys or materials can be used to create interactive and immersive learning experiences when paired with an RFID reader-embedded table or display. Furthermore, RFID technology has been studied for indoor localization of robot agents, and the current method can help identify the direction in which the agent is heading, improving overall agent localization (Zhang et al., 2023).

The current study validated the concept with a small number of healthy adults. The results showed equivalent sensor performance across demographic factors, but further investigation is required to assess feasibility in the target population of younger children with smaller body/head size. Although this study has assumed a scenario where the wearer is watching media on a screen, the requirement that the wearer remain seated stationary to ensure performance is a significant limitation of this sensor. Achieving stable reading of passive RFID tag sensors remains a significant challenge due to the dependence of RF signal strength on the surrounding environment. To eliminate weak signals coming through multi-paths and exclude interference from obstacles, other RFID tags, or metal, the study assumed a short distance between the reader and tag. However, the presence of these factors can significantly impact sensor functionality.

Currently available portable devices in the market, such as smartphones or laptops, only have an RFID reader functioning at a lower frequency (HF – high frequency, NFC – near field communication). RFID reader-embedded devices operating at UHF are promising for larger detection distances and more versatile applications for everyday sensing. RF exposure to human tissue is another important factor that must be carefully designed for the safety of the user, especially when the reader is close to the body. The U.S. Federal Communications Commission (FCC) requires the Specific Absorption Rate (SAR) to be less than 1.6 W/kg over 1 g of human tissue, and the transmitting power of the RFID reader used in this study is 32 dBm (1.58 W), which is significant at close distances. Therefore, the transmission power of the RF signals must be carefully adjusted to ensure the safety of users and the optimal and stable performance of the sensor.

## 5. Conclusion

This study introduces a battery-free wearable head orientation sensor using passive RFID tags. The RSSI of the RFID tags changes based on the distance to the reader, which depends on the head rotation in yaw or pitch. Preliminary lab tests have confirmed that settings such as 13 cm between tags, 50 cm from the reader, and PET-based tag can generate optimal performance. Testing with human participants showed that the RSSI difference between the tags correlated with head orientation data collected by a computer vision-based system, especially when the wearer continuously and randomly rotated their head rather than freely looking at the laptop. However, the sensor could not track pitch rotation as well as the wearer’s gaze, which remains as a possibility for expansion of the current study.

## Data Availability

The data that support the findings of this study are available on request from the corresponding author, H.T.P. The data are not publicly available due to their containing information that could compromise the privacy of research participants.
